# Sleep Bruxism and Orofacial Pain in Patients with Sleep Disorders: A Controlled Cohort Study

**DOI:** 10.3390/jcm12082997

**Published:** 2023-04-20

**Authors:** Maria Lavinia Bartolucci, Serena Incerti Parenti, Francesco Bortolotti, Veronica Della Godenza, Stefano Vandi, Fabio Pizza, Giuseppe Plazzi, Giulio Alessandri-Bonetti

**Affiliations:** 1 Department of Biomedical and Neuromotor Sciences, University of Bologna, 40125 Bologna, Italyfrancesco.bortolott4@unibo.it (F.B.);; 2 IRCCS Istituto delle Scienze Neurologiche di Bologna, 40139 Bologna, Italy; 3 Department of Biomedical, Metabolic and Neural Sciences, University of Modena and Reggio Emilia, 41125 Modena, Italy

**Keywords:** sleep bruxism, temporomandibular disorders, tooth wear, sleep disorders, TMD, temporomandibular joint

## Abstract

Background: The gold standard for the diagnosis of sleep bruxism (SB) is laboratory polysomnography (L-PSG) recording. However, many clinicians still define SB using patients’ self-assessment and/or clinical tooth wear (TW). The purpose of this cross-sectional controlled study was to compare the prevalence of TW, head-neck muscles sensitivity and Temporomandibular Disorders (TMD) between SB and non-SB patients diagnosed with L-PSG in a cohort of patient with sleep disorders (SD). Methods: 102 adult subjects with suspected SD underwent L-PSG recording to assess the presence of sleep disorder and SB. TW was clinically analyzed using TWES 2.0. The pressure pain threshold (PPT) of masticatory muscles were assessed using a Fisher algometer. Diagnostic criteria for TMD (DC/TMD) were used to evaluate the presence of TMD. SB self-assessment questionnaires were administered. TWES score, PPT, TMD prevalence and questionnaire results were compared between SB and non-SB patients. Results: 22 SB patients and 66 non-SB patients with SD were included. No significant differences emerged between groups in regards to TW, the PPT values, or SB’s self-assessment questionnaires as well the prevalence of TMD. Conclusion: in a SD population, TW is not pathognomonic of active SB and SB self-assessment is not reliable. There seems to be no correlation between SB, TMD and head/neck muscle sensitivity.

## 1. Introduction

In recent years, a remarkable effort has been made toward the refinement of the definition and classification of bruxism. Bruxism is described as a repetitive jaw-muscle activity characterized by clenching or grinding of the teeth and/or by bracing or thrusting of the mandible [1]. It is a complex condition with a multifactorial origin: biological characteristics, environment, genetics and lifestyle seem to play a role [2]. Bruxism has two distinct circadian manifestations: awake bruxism (AB), characterized by prolonged or repetitive tooth contact and/or by bracing or thrusting the mandible, and sleep bruxism (SB), characterized by a rhythmic (phasic), non-rhythmic (tonic) or mixed (phasic and tonic) muscle activity [1]. The prevalence of self-reported SB is 12% in the general adult population, with a maximum peak between 20 and 40 years of age and a tendency to decrease with age [3]. In healthy individuals, SB is not considered a sleep disorder; instead, it is considered a movement behavior and its occurrence has been correlated with central neurotransmission system disorders [1,4]. Some evidence shows that anxiety, depression and stress [5,6] can increase SB; SB also seems to be influenced by smoking habits, alcohol assumption, caffeine and overuse of other stimulants [7]. 

SB has been associated with several clinical consequences, including dental hard tissues damage (e.g., cracked teeth), mechanical tooth wear, masticatory muscle hypertrophy, indentations on the tongue or lip [1] and repetitive failures of restorative therapies [8,9]. However, detecting signs of attrition is not pathognomonic of a current activity. Many factors can contribute to tooth wear, including mechanical abrasion and/or chemical erosion, alongside physiological tissue loss [1,10,11]. The literature does not provide parameters on the factual involvement of bruxism on tooth wear. Likewise, there is no consensus on a possible connection between SB and Temporomandibular Disorders (TMD); TMDs are highly prevalent musculoskeletal and neuromuscular conditions that affect the temporomandibular joint, masticatory muscles and associated structures [12,13]. Among the proposed treatments, [14,15,16,17], behavioral and physical therapy have been shown to be the most appropriate and effective for the management of TMD [18].

Both SB and TMD are made worse by anxiety, depression and stress [5,19,20]. Several patients report masticatory muscles pain/fatigue in the morning; however, a recent scoping review [21] highlights that it is not possible to draw conclusions on a real cause-effect relationship between SB and TMD. This issue is partially due to the lack of homogeneity in the evaluation methods used in the investigations. Although laboratory Polysomnography (L-PSG) is the gold standard for the diagnosis [22], it may not be sufficient to perform the assessment with just a contingent recording since bruxism is an activity that fluctuates over time. 

L-PSG is the reference tool for studying sleep and its influence on physiologic functions. It is essential for the diagnosis of sleep disorders, functioning by carrying out a simultaneous registration of electroencephalography (EEG), eye movements by electrooculogram, chin and limbs electromyography (EMG), respiratory air flow, arterial oxygen saturation and electrocardiogram (ECG) in a laboratory setting with a technician constantly in attendance and responsible for the correct execution of the study. Moreover, audiovisual recordings are performed during the examination to enhance diagnostic power. Manual scoring of the registrations is performed by experts to define sleep stages and possible events.

The instrumental data, as suggested by some authors, could benefit from association with clinical evaluations and from self-reported information that, alone, would result non-reliable and inaccurate due to the lack of patients’ awareness of oral behaviors [21]. 

The purpose of the present controlled cohort study was to compare the clinical parameters of tooth wear amount, pressure pain thresholds (PPT) of head and neck muscles, TMD prevalence and the results of SB self-assessment questionnaires between a cohort of patients affected by SB and a control group. 

## 2. Materials and Methods

The present study was approved by the Ethics Committee of the Area Vasta Emilia Centro of the Emilia-Romagna Region (CE-AVEC), with the number EM297-2021-19080-EM1-OSS-AUSLBO. 

### 2.1. Population

A controlled cohort study was conducted on subjects referred to the Center for the Study and Treatment of Sleep Disorders of the Bellaria Hospital in Bologna, with suspected of sleep disorders. 

Inclusion criteria were a minimum age of 18 years and the diagnosis of a sleep disorder. Patients taking non-steroidal anti-inflammatory drugs, paracetamol and opioid analgesics in the previous 5 days, steroidal drugs in the previous 30 days, anti-depressants, membrane-stabilizing drugs, and oral contraceptives [23], subjects suffering from painful acute oral diseases (e.g., pulpitis, dental fractures), cervical spine dysfunction, affected by diabetes and non-self-sufficient individuals (necessitating material and psychological support due to physical impairment or previous accidents) were excluded from the present study. 

### 2.2. Procedure

All the subjects underwent the following standardized procedures. A clinical neurological evaluation was performed by an expert in sleep medicine who also administered the Epworth Sleepiness Scale (ESS) [24], to assess the subjects’ sleepiness, and the Pittsburg Sleep Quality Index (PSQI) [25]. As indicated by the American Academy of Sleep Medicine (AASM), L-PSG recordings were carried out for 3 consecutive nights to improve statistical validity. Recordings began the night following the patient’s hospitalization to allow for adaptation to the new environment. 

The PSGs were carried out in a dark, soundproofed and temperature-controlled room and included conventional EEG, EMG of the right masseter, the right and left submental muscles and the right and left Tibialis muscles, ECG, bilateral electrooculogram, respiratory monitoring, pulse oximetry and audio/video recordings. The montage of the electrodes was performed following AASM guidelines and those of the American Association of Sleep Technologists for standard polysomnography [26].

EMG was performed by fixing sensors to the skin in a non-invasive manner. The recordings of submental muscle were used to determine the level of muscle tone, which gradually decreases as one progresses through the deeper stages of sleep, as well as for registering bruxism activity. An additional electrode was placed on the masseter muscle to better determine bruxism episodes. The guidelines recommend including this additional electrode as a separate recording channel. The L-PSG used in the study presented one more available channel that was used for the masseter muscle analysis. 

Following the AASM guidelines, one electrode was placed 1 cm lateral and 1 cm above the right outer canthus and another electrode was placed 1 cm lateral and 1 cm below the left outer canthus to register electrooculogram. The EMG of the submental muscle was registered by placing one electrode in the midline, 1 cm above the inferior edge of the mandible, one electrode 2 cm below the inferior edge of the mandible and 2 cm to the left of the midline and another one placed 2 cm below the inferior edge of the mandible and 2 cm to the right of the midline. To evaluate the masseter muscle, the electrode was placed on the jaw line. The monitoring of anterior tibialis muscles was performed by placing the surface electrodes longitudinally and symmetrically, lengthwise, in the center of the muscle. The electrodes remained in place for the entire duration of hospitalization to collect information about the extension, strength and duration of muscle activity. Before starting the sleep recording, a calibration test was performed in order to assess baseline values for each parameter (e.g., limb movements, swallowing, maximum voluntary eye movements). To calibrate the masseter’s EMG signal, the subject was required to clench the teeth for 2 s while a signal was recorded at 512 Hz and filtered (hardware: notch at 50 Hz; high pass at 10 Hz; low pass at 100 Hz). The PSG recordings were analyzed using DOMINO Sleep Diagnostic software (Somnomedics, Randersacker, Germany). A semi-automatic SB analyzing tool incorporated in the DOMINO software was used for the pre-investigation analysis of events. The SB diagnostic cut-offs set in the software are defined following those described by Lavigne et al. [22] In accordance with AASM guidelines [26], the sleep analyses were performed over 30 s epochs and were directed towards a series of specific parameters: sleep onset latency from lights off, REM sleep latency from sleep onset, wakefulness after sleep onset, total sleep time (TST), sleep period from sleep onset to lights on, sleep efficiency, percentage of time spent in each sleep stage, awakenings, awakenings per hour, respiratory disturbance index, apnea/hypopnea index and periodic limb movement index. Analysis of the EMG signal was carried out by an operator trained in the procedure and with expertise in the diagnosis of SB; this operator manually checked all the tracings to include only the rhythmic masticatory muscles activities (RMMA) performed during actual sleep. According to the Kondo and Clark criteria [27], a threshold of 20% of the maximum voluntary EMG contraction of the masseter muscle was used to detect the RMMA. EMG activities characterized by one burst lasting more than 2 s or by at least 3 bursts with a duration between 0.25 and 2 s, presenting an inter-burst interval less than 3 s, were indicated as SB episodes [22]. 

In order to enroll a homogeneous sample, only the subjects who received a diagnosis of sleep disorder after the PSG recordings were included in the present study. Based on PSG results, two groups were formed: SB patients and non-SB patients (controls). 

All patients were examined by the same operator, with expertise in orofacial pain, following the Diagnostic Criteria for Temporomandibular Disorders (DC/TMD) [28] including a physical examination using reliable and well-operationalized diagnostic criteria (AXIS I) and an evaluation of psychological status and pain-related disability (Axis II). The patients received the Graded Chronic Pain Scale (GCPS) [29] to describe pain intensity and pain-related disability, the Jaw Functional Limitation Scale (JFLS) [30] to evaluate the functional status of the masticatory system, the Patient Health Questionnaire-9 (PHQ-9) [31] to assess psychological distress due to depression, the Generalized Anxiety Disorder-7 (GAD-7) [32], the Physical symptoms questionnaire (PHQ-15) [33] and the Oral Behaviors Checklist (OBC) [34] investigating the frequency of oral parafunctional habits. Moreover, a SB self-assessment questionnaire [35] was administered to all subjects.

The pressure pain thresholds (PPT) of head and neck muscles, defined as the lowest pressure that induces pain or discomfort [36], were evaluated in order to detect possible differences between groups.

**Figure 1:**
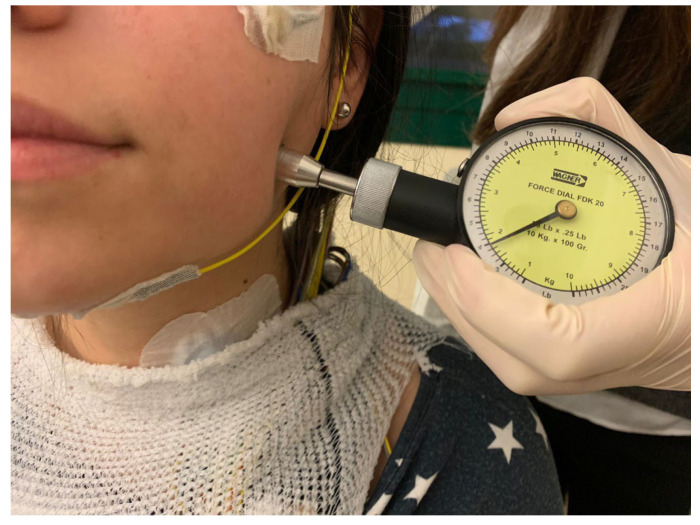
PPT registration on the left masseter muscle.

A calibrated examiner, who was blind to the subject’s group, performed the measurements bilaterally on the temporalis (anterior, middle, posterior), masseter, sternocleidomastoid, occipital and splenius capitis muscles using a Fisher algometer with a standard rate of pressure increase of 100 g/sec [37]. The tests were carried out with the subject in a standardized natural position: sitting with their back at 90° to the floor, teeth not in contact and muscles relaxed. The patient was instructed to raise the left hand when the minimum pain/discomfort sensation (threshold) was reached (Figure 1).

The presence of tooth wear was clinically determined through an intraoral examination using the 5-point grading scale “TWES 2.0 tooth wear evaluation system” for each tooth. This grading system defines the severity of tooth wear using an ordinal scale with a score from 0 to 4 points for the occlusal/incisal surfaces: 0 indicates no wear, 1 indicates visible wear limited within the enamel, 2 indicates wear with dentin exposure of less than 1/3 of the height of clinical crown, 3 indicates visible wear with dentin exposure and loss of clinical crown height of more than 1/3 but less than 2/3 and 4 indicates visible wear with dentin exposure and loss of more than 2/3 of the height of the clinical crown [38,39]. The operator used a pcp-unc15 periodontal probe (Hu-Friedy Italy, Milano, Italy) to standardize the evaluation. 

### 2.3. Statistical Analysis

The sample size calculation was performed considering the difference in pressure pain thresholds of the masseter muscle between the SB group and the control group as the primary outcome, setting the alpha error at 0.05 and the beta error at 0.20. Setting the effect size at 0.7, a minimum sample size of 88 subjects is required. After verifying the normal distribution of the data, the comparison of the PPT between the two groups was carried out with the t test for independent samples. The prevalence of TMD diagnoses (Axis I) and the results of the questionnaires (Axis II) in terms of cut-off between the 2 groups were compared using the χ^2^ test. This test was also used to compare the differences of clinical tooth wear between the two groups. The TWES median score per participant was used as a summary measure of tooth wear and it was compared between groups using the Mann–Witney test.

## 3. Results

Starting from 108 subjects, 88 patients were included in the present study: 30 patients affected by type 1 Narcolepsy, 11 affected by type II Narcolepsy, 24 with Hypersomnia, 6 with Periodic Limb Movement, 6 affected by Obstructed Sleep Apnea (OSA), 6 with Parasomnias and 5 with REM Behavior Disorder. The SB group was composed of 22 subjects who tested positive for SB (14 males and 8 females, mean age of 31.7 ± 15.4 years) by means of the L-PSG. The non-SB group was the control group, made up of 66 subjects (32 males and 34 females, mean age of 36.4 ± 13.9 years) who tested negative for SB. Table 1 presents the demographic characteristics of the two groups, the results of the ESS, of the PSQI, of the SB self-assessment questionnaire and of the sleep analysis. 

**Figure 2:**
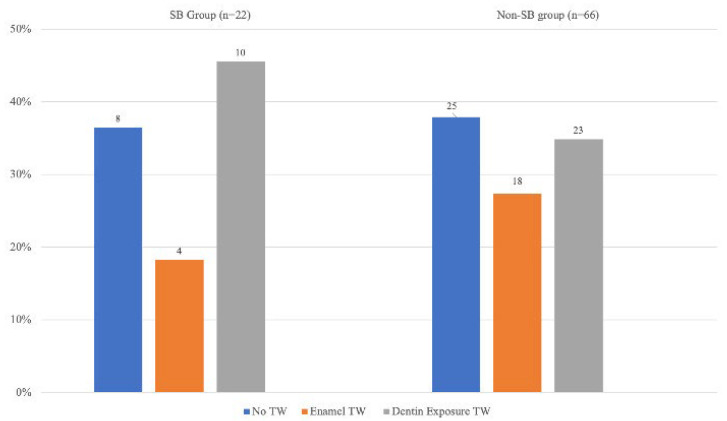
TWES 2.0 results in SB and Non-SB groups.

Concerning the sleep parameters, the mean value of the three recordings was computed. No statistically significant differences emerged between the SB group and non-SB group for all the variables analyzed. Figure 2 shows the differences in clinical tooth wear between the two groups: the subjects were divided into three categories corresponding to absence of tooth wear, tooth wear limited to the enamel layer and tooth wear with dentin exposure. 

No significant differences were detected between the two groups. Table 2 shows the PPTs of the subjects: right and left values were compared by means of the t test; since no statistically significant differences were detected, data from right and left muscles were merged for the statistical analysis. No significant difference in the PPT values of all muscles examined were registered between the SB group and non-SB group. 

No significant differences emerged between the two groups in the prevalence of TMD diagnoses nor in the results of the axis II questionnaires (Table 3). 

## 4. Discussion

The present study was aimed at evaluating possible differences in clinical tooth wear, SB self-assessment questionnaire scores, PPT of head and neck muscles and TMD diagnoses between one group of SB patients and one group of non-SB patients who were selected by means of L-PSG from a cohort of sleep disorder patients. The absence of significant differences between the two groups in the sleep parameters that were analyzed and in their demographic characteristics (Table 1) underline the homogeneity of the sample. The main outcome is represented by the absence of significant differences between the SB group and the non-SB group in relation to all the parameters evaluated. Considering the self-assessment questionnaire, the results of the present study are in accordance with data presented in the literature [11], showing a low reliability of the tool: no differences emerged in SB self-reporting between SB and non-SB subjects. As far as the tooth wear is concerned, the absence of a significant difference between the two groups is consistent with the current evidence supporting a “multifactorial” etiology underlying the tissue loss. It should be regarded as the result of different interactions between physiological functional wear, chemical erosion and intrinsic enamel characteristics, not just as a reliable indicator of active bruxism [1,10,11]. It is also very important to underline and discuss the absence of differences between the two study groups in regards to the evaluated muscle PPT and in the prevalence of TMD. No differences in the PPT of head and neck muscles emerged; this was consistent with a previous investigation that compared patients affected by sleep breathing disorders (OSA) and healthy subjects [40]. The correlation between SB and TMD has been highly debated and the literature provides differing points of view and results. Among the papers supporting this correlation, some studies present selection bias, indicating, as one of the inclusion criteria, the self-reporting of signs and symptoms of pain and/or considering the presence of wear on anterior teeth as a risk factor for TMD onset [41]. Other authors did not find evidence for the association between SB and TMD, therefore suggesting a cautious approach in this regard [21]. The present study performed the assessments by means of the DC/TMD protocol; this protocol is considered to be the gold standard for TMD diagnosis. The study did not find significant differences between the groups in the presence of TMD, both of muscular and articular origin. This outcome is consistent with the scores of Axis II questionnaires, showing no difference in psycho-social comorbidities between the groups. Some studies, investigating the association between bruxism and psychological distress, suggested that AB appears to be associated with psychosocial factors and a range of psychopathological symptoms, while there is no evidence to relate SB to psychosocial disorders [6]. Moreover, the analysis of the OBC questionnaires supports the results on the absence of differences in tooth wear between the study groups and discards the hypothesis of a possible relationship between oral behaviors and TMD [4]. It is interesting to note that studies based on clinical diagnosis of SB or on SB self-assessment questionnaires reported a positive association with the presence of orofacial pain [41,42,43,44], while PSG studies found a weak association or even a negative relationship between SB and TMD. These different outcomes are linked to the methodology applied and represent the fulcrum of the most recent disquisitions by leading authors in this field [21,45]. In fact, Manfredini and coworkers underline the reliability of PSG in providing an objective quantification of the SB events but also support the need to approach SB as a complex condition that requires interpretation and, therefore, a concurrent clinical evaluation with specific protocols [46]. The results of the present study underline the poor reliability of the clinical evaluation and of the self-assessment questionnaire to make a diagnosis of SB and support L-PSG as the diagnostic gold standard [1,22]. It is not a routine examination due to its complexity, high cost, burden and discomfort for the patient and it is normally used for the diagnosis of sleep disorders endangering patients’ life. Therefore, it is very difficult to carry out a polysomnographic study aimed at evaluating SB on healthy patients. In this study, the sample was selected from patients with sleep disorders who were included in a standardized diagnostic protocol that consisted of a clinical evaluation performed by the same specialists and in a laboratory analysis carried out with the same recording instruments and L-PSG for three consecutive days. Even if not strongly supported by the literature [47,48] a night-to-night variability has been described in patients with SB [49]; the study setting allowed for a more reliable evaluation, reporting the average of the events per subject. The two groups were formed after the L-PSG scoring that consisted of an analysis of the raw tracings of EEG and EMG and of the video recording. The study sample was not screened for the presence of AB. This activity has been associated with psychological distress and, in some individuals, it could bring about muscle pain conditions [50,51]. To this regard, having not excluded AB could be a confounding factor and could overlap with the clinical signs and symptoms being assessed. Some authors have reported a greater reliability of the bilateral electrode on the masseter muscle in determining SB episodes but also report a similar sensitivity to the single electrode [52]. This possible limitation is due to the characteristics of the instrument used to perform the L-PSG in the present study setting; however, it should be taken into consideration that the manual review of the video in correspondence of each single event reinforces the results. The present outcomes suggest defining SB as an unconscious motor event with a multifactorial etiology and not necessarily related to pain, tissue damage and dysfunction. Given the difficulty of performing L-PSG in clinical settings and considering the large number of possible variables that could influence SB with different manifestations, further research should focus on evaluating the specific and sustainable clinical diagnostic tools that have been recently presented in studies on both healthy and SD patients [46,53,54].

## 5. Conclusions

In the sample evaluated, the presence of SB did not show significant correlation with the presence of tooth wear, the self-perception of the activity and the presence of TMD. No differences emerged concerning the bio-psycho-social parameters between the two groups.

## Figures and Tables

**Table 1:** Sample description. Data are reported as mean ± standard deviations or number of subjects and percentage. * = significant difference between groups.

	SB (n = 22)	Non-SB (n = 66)	*p*
Age	31.73 ± 15.41	36.44 ± 13.94	0.185
Gender	14 M (63.6%), 8 F (36.4%)	32 M (48.5%), 34 F (51.5%)	0.218
ESS	11.36 ± 5.50	11.66 ± 5.26	0.826
PSQI	7.86 ± 3.60	7.61 ± 4.03	0.800
Self-Reported SB Questionnaire	12 SB (54.5%), 10 non-SB (45.5%)	25 SB (37.9%), 41 non-SB (62.1%)	0.170
TST (in hours)	7.08 ± 1.09	7.68 ± 5.40	0.607
TST N1 (%)	7.05 ± 4.44	8.02 ± 6.94	0.541
TST N2 (%)	42.57 ± 9.77	39.96 ± 10.62	0.311
TST N3 (%)	6.12 ± 2.27	7.54 ± 3.20	0.058
TST N4 (%)	22.15 ± 9.50	22.41 ± 8.92	0.908
TST REM (%)	21.81 ± 5.64	21.27 ± 6.21	0.719
Sleep Efficiency	85.84 ± 8.84	83.48 ± 12.57	0.418
Bruxism Episodes per hour	4.98 ± 2.48	0.77 ± 0.74	0.001 *

TST = total sleep time, REM = rapid eye movement.

**Table 2:** PPT (mean ± SD) recorded in the two groups (kg/cm^2^) and comparisons between the groups (t test).

Muscles	SB (n = 22)	Non-SB (n = 66)	t
Anterior Temporal	2.90 ± 0.96	2.66 ± 0.89	−1.089
Middle Temporal	3.36 ± 0.94	2.98 ± 0.95	−1.640
Posterior Temporal	3.73 ± 1.05	3.28 ± 1.05	−1.742
Masseter	2.23 ± 1.04	1.96 ± 0.74	−1.343
Sternocleidomastoid	1.84 ± 0.64	1.62 ± 0.66	−1.249
Occipital	2.89 ± 1.04	2.67 ± 1.02	−0.868
Splenius capitis	2.48 ± 0.94	2.24 ± 0.94	−1.052
Thenar	3.99 ± 1.10	3.96 ± 1.57	−0.075

**Table 3:** Comparison of Prevalence of TMD diagnoses (Axis I) and prevalence of over cut-off scores of the Axis II DC/TMD questionnaires between groups (χ^2^ test). Prevalence is reported as percentage and number of subjects.

	SB (n = 22)	Non-SB (n = 66)	χ^2^	*p*
TMD	8 (36.4%)	21 (31.8%)	0.154	0.694
Muscle TMD	7 (31.8%)	22 (33.3%)	0.017	0.896
Articular TMD	3 (13.6%)	17 (25.8%)	1.380	0.240
GCPS 2.0 (Chronic Pain)	5 (22.7%)	20 (30.3%)	0.466	0.495
JFLS-20 (Functional limitation)	4 (18.2%)	18 (27.3%)	0.727	0.394
PHQ-9 (Depression)	51 (77.3%)	14 (63.6%)	1.589	0.207
PHQ-15 (Physical symptoms)	14 (63.6%)	46 (69.7%)	0.279	0.597
GAD-7 (Anxiety)	10 (45.5%)	36 (54.5%)	0.547	0.460
OBC (Oral parafuntions)	9 (40.9%)	32 (48.5%)	0.381	0.537

## Data Availability

The data presented in this study are available on request from the corresponding author.

## Abbreviations

SB: sleep bruxism; L-PSG: laboratory polysomnography; TW: tooth wear; SD: sleep disorders; TMD: temporomandibular disorders; PPT: pressure pain thresholds; DC/TMD: diagnostic criteria for TMD; AB: awake bruxism; ESS: Epworth sleepiness scale; PSQI: Pittsburgh Sleep Quality Index; EEG. Electroencephalography; EMG: electromyography; ECG: electrocardiogram; TST: total sleep time; RMMA: rhythmic masticatory muscle activity, TWES: tooth wear evaluation system; GCPS: graded chronic pain scale; JFLS: jaw functional limitation scale; PHQ-9: patient health questionnaire-9; PHQ-15: patient health questionnaire-15; GAD-7: generalized anxiety disorder; OBC: oral behavior checklist.
